# Is digital photography an accurate and precise method for measuring range of motion of the hip and knee?

**DOI:** 10.1186/s40634-017-0103-7

**Published:** 2017-09-07

**Authors:** Russell R. Russo, Matthew B. Burn, Sabir K. Ismaily, Brayden J. Gerrie, Shuyang Han, Jerry Alexander, Christopher Lenherr, Philip C. Noble, Joshua D. Harris, Patrick C. McCulloch

**Affiliations:** 1 Department of Orthopedics & Sports Medicine, Houston Methodist Hospital, 6445 Main Street, Outpatient Center, Suite 2500, Houston, TX 77030 USA; 2 Institute for Orthopaedic Research & Education (IORE), Houston, TX USA

**Keywords:** Hip, Knee, Range of motion, Digital photography, Goniometry, Visual estimation

## Abstract

**Background:**

Accurate measurements of knee and hip motion are required for management of musculoskeletal pathology. The purpose of this investigation was to compare three techniques for measuring motion at the hip and knee. The authors hypothesized that digital photography would be equivalent in accuracy and show higher precision compared to the other two techniques.

**Methods:**

Using infrared motion capture analysis as the reference standard, hip flexion/abduction/internal rotation/external rotation and knee flexion/extension were measured using visual estimation, goniometry, and photography on 10 fresh frozen cadavers. These measurements were performed by three physical therapists and three orthopaedic surgeons. Accuracy was defined by the difference from the reference standard, while precision was defined by the proportion of measurements within either 5° or 10°. Analysis of variance (ANOVA), t-tests, and chi-squared tests were used.

**Results:**

Although two statistically significant differences were found in measurement accuracy between the three techniques, neither of these differences met clinical significance (difference of 1.4° for hip abduction and 1.7° for the knee extension). Precision of measurements was significantly higher for digital photography than: (i) visual estimation for hip abduction and knee extension, and (ii) goniometry for knee extension only.

**Conclusions:**

There was no clinically significant difference in measurement accuracy between the three techniques for hip and knee motion. Digital photography only showed higher precision for two joint motions (hip abduction and knee extension). Overall digital photography shows equivalent accuracy and near-equivalent precision to visual estimation and goniometry.

## Background

When assessing hip and knee pathology, range of motion (ROM) is a commonly used clinical parameter utilized by medical professionals. Accurate measurements of ROM are important for diagnosis, monitoring progression or resolution of symptoms, clinical decision-making, surgical planning, assessing treatment response, for research, and to evaluate permanent disability or impairment (Lavernia et al. 2008; Lea and Gerhardt 1995; Mai et al. 2012). In addition, it allows the patient to appreciate their own progress during clinical visits and can be used as goals for rehabilitation (e.g. knee flexion needed to ascend and descend stairs) (Brosseau et al. 2001; Lavernia et al. 2008). Within orthopaedic surgery, accurate measurement of hip and knee ROM is critical for assessing the outcomes of surgery.

The two most commonly used techniques for assessing range of motion are visual estimation and goniometry (Chevillotte et al. 2009; Ferriero et al. 2013; Gajdosik and Bohannon 1987; Lavernia et al. 2008; Murphy et al. 2013). Of these, goniometry is often believed to offer more accurate and reliable measurements than visual estimation (Brosseau et al. 2001; Chevillotte et al. 2009; Edwards et al. 2004; Ferriero et al. 2013; Gajdosik and Bohannon 1987; Herrero et al. 2011; Holm et al. 2000; Lavernia et al. 2008; Lea and Gerhardt 1995; Murphy et al. 2013; Roach et al. 2013; Watkins et al. 1991). However, it requires two hands for use (leaving neither hand free for limb stabilization) and more time than visual estimation (Nussbaumer et al. 2010). Many other less commonly utilized techniques have been studied within the literature (Charlton et al. 2015; Chevillotte et al. 2009; Herrero et al. 2011; Holm et al. 2000; Lea and Gerhardt 1995; Roach et al. 2013). The accuracy and reliability of any of these techniques has been shown to improve with repeated measurements, either by different investigators or the same investigator multiple times (Boone et al. 1978; Edwards et al. 2004; Watkins et al. 1991). Digital photography offers additional benefits as it allows for comparison between observations of the same measurement, different measurements separated by time, and allows for off-site measurements over long distances (such as for telemedicine or internet-based healthcare) (Naylor et al. 2011). Smartphone technology, which has become almost universally available, facilitates this technique (Charlton et al. 2015; Chevillotte et al. 2009; Herrero et al. 2011; Murphy et al. 2013; Naylor et al. 2011; Russell et al. 2003; Verhaegen et al. 2010).

The purpose of this study was to compare the accuracy of ROM measurements of hip and knee motion using multiple techniques (visual estimation, goniometric measurement, and digital photographic measurement). The authors hypothesized that digital photography would be equivalent in accuracy and show higher precision compared to the other two techniques.

## Methods

Using G*Power software (Universität Mannheim, Mannheim, Germany) and assuming mean measurement error of 3° ±5° (effect size 0.6) between each of the measurement techniques (goniometry, digital photography, visual estimation), an a priori power analysis (β = 0.20, α = 0.05) predicted that we would require 45 measurements with each of the three techniques. This requirement would be met with 3 investigators each taking measurements on 16 lower extremities [8 cadavers] with each of the 3 techniques, however it was decided to include 6 investigators from two different specialties (orthopaedic surgery and physical therapy) to broaden the scope and generalizability (Faul et al. 2009; Faul et al. 2007).

After institutional review board (IRB) approval, ten fresh-frozen human cadavers were obtained without specifying race, gender, ethnicity, age, or cause of death. The only exclusion criteria were gross limb deformity or amputated limbs. All specimens were stored at −5 °C and thawed 24 h prior to testing. Ten cadavers were used (20 lower extremities, measured by six investigators using three different techniques, 120 measurements by each technique) for measurements in two different sessions (5 different cadavers were used in each session) separated by a two-month period. For each of the two sessions, the five cadavers used were not refrozen after initial thawing and thus all measurements were obtained over a 3-day period.

Three of the investigators were licensed physical therapists (PT) with greater than 6 months of clinical experience and three were board-certified fellowship-trained orthopaedic surgeons. The orthopedic surgeons included two sports medicine fellowship trained surgeons and one adult reconstructive fellowship trained surgeon. All investigators took measurements of six selected motions (hip flexion, hip abduction, hip internal rotation, hip external rotation, knee flexion, and knee extension) using three techniques (visual estimation, goniometric measurement, and digital photographic measurement) on each cadaveric specimen bilaterally (both lower limbs).

### Cadaver & Motion Analysis Setup

**Fig. 1:**
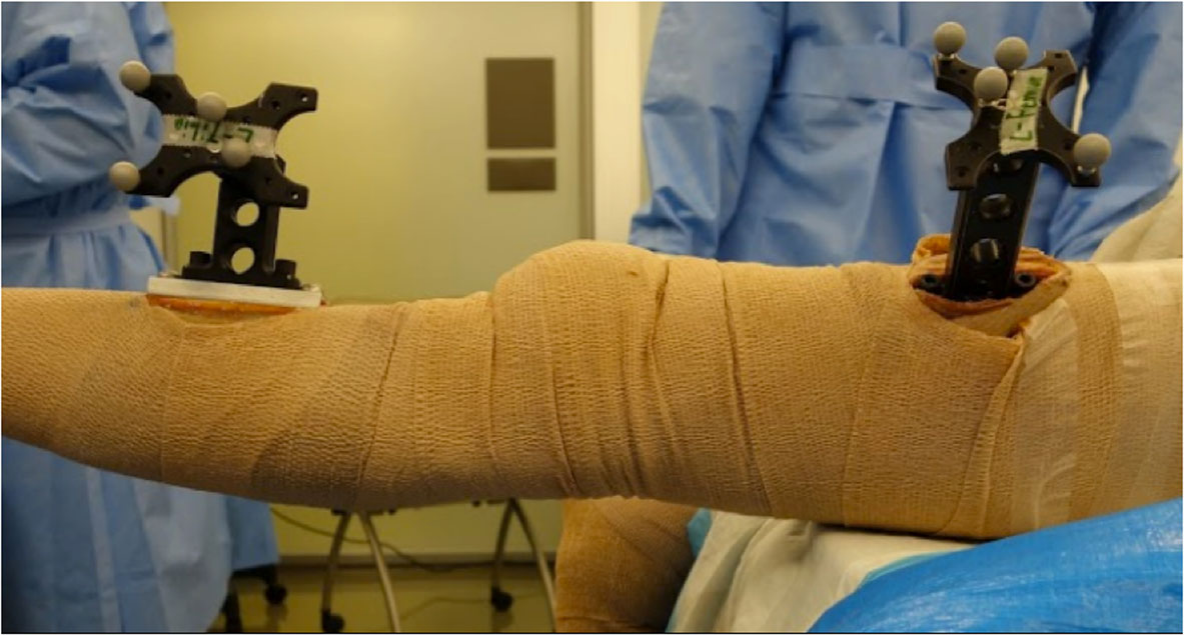
Photograph demonstrating the arrays of reflective markers used for infrared motion capture analysis, which are fixed to the femur (right) and the tibia (left) using a combination of screw fixation and bone cement

Prior to beginning each measurement session, specific sites on each of the five cadavers (to be used for that session) were dissected down to bone bilaterally where mounting plates were secured rigidly with screw fixation and cementation (using polymethyl methacrylate) to three sites. The three mounting sites used bilaterally, included (1) the iliac crest, (2) the anterolateral aspect of the femoral midshaft, and (3) the anterior aspect of the tibial midshaft. Arrays of reflective markers (NDI, Waterloo, Canada – shown in Fig. 1) including passive reflective spheres were attached to each mounting site to track three-dimensional (3D) spatial location of each of these bones during the measurement session.

Prior studies have used radiographic (two-dimensional) measurements as their “gold standard” with which to compare other measurements (Chapleau et al. 2011). Computed tomography (CT)-based motion analysis offers an additional advantage of being able to measure the joint angle in three-dimensions and being able to account for rotation (e.g. measurement of elbow flexion with differing humeral rotation). To define the “gold standard” used for this study (motion capture analysis), the lower extremities of all 10 cadavers underwent computer tomographic (CT) scans with mounting sites and markers attached. This Digital Imaging and Communications in Medicine (DICOM) data was used to construct three-dimensional (3D) models of each joint to be measured with software from Materialise Mimics (Materialise, Leuven, Belgium). Each 3D model was imported into Rapidform (INUS Technology Inc., Seoul, Korea) to be used with the motion capture device in combination with a custom MATLAB program (The MathWorks Inc., Massachusetts, USA). This process allowed for accurate joint angle calculations to be performed in real-time during measurement sessions.

**Fig. 2:**
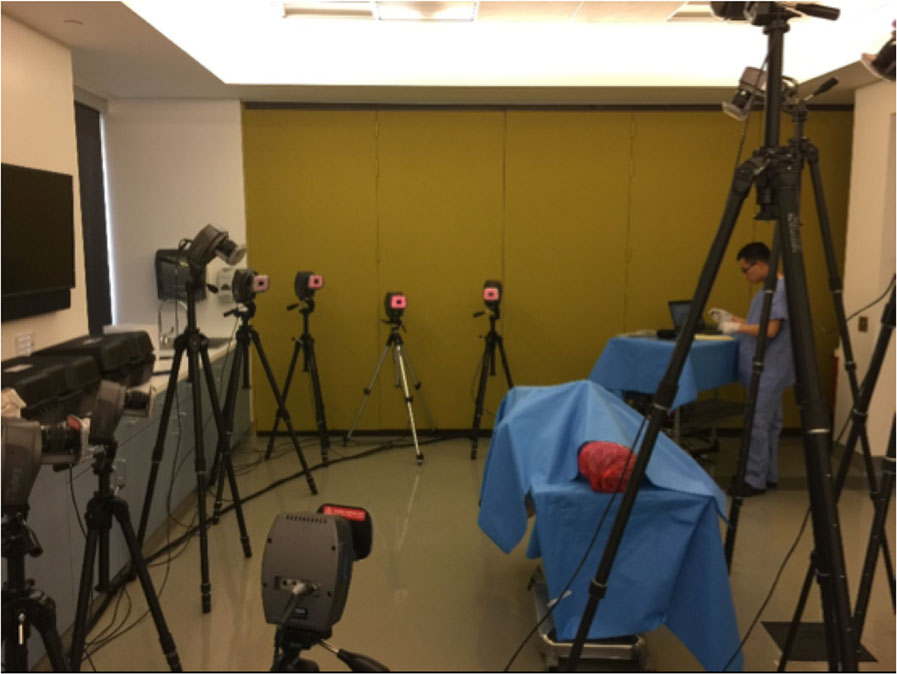
Room set up for the measurements. An operating room (OR) table was positioned in the center of twelve motion analysis cameras on tripods at different heights and angles. These cameras were pre-calibrated prior to each measurement session

Arrays of markers, but not the mounting plates, would be rearranged once each joints’ angle was to be measured. For example, when testing knee flexion and extension, the markers would be attached to the femur and tibia on one side of the body only. Markers would be removed from the contralateral lower extremity. This was done to aid the accuracy of motion analysis by avoiding confusion of the twelve motion analysis cameras (Motion Analysis, Santa Rose, CA), which were set up in a semi-circle surrounding an operating room (OR) table holding the cadaver (Fig. 2). The position of the table and cameras were calibrated prior to beginning a measurement session and remained constant for all investigators’ measurements. Clear visualization of the arrays by at least two cameras simultaneously is the minimum requirement for accurate localization, but this study used a minimum of three cameras to guarantee accuracy (Furtado et al. 2013).

### Measurement technique

During measurement sessions, each of the cadavers, one at a time, was positioned supine on an operating room (OR) table in the center of the twelve calibrated motion analysis cameras (Fig. 2). A single assistant () would position the limb at the maximum joint motion and hold it in place for all measurements. First, the exact skeletal location (joint angle) would be calculated by computer-assisted infrared camera motion capture analysis (Furtado et al. 2013). This would establish the gold standard measure for comparison by this investigator of this joint motion to all three other techniques.

**Fig. 3:**
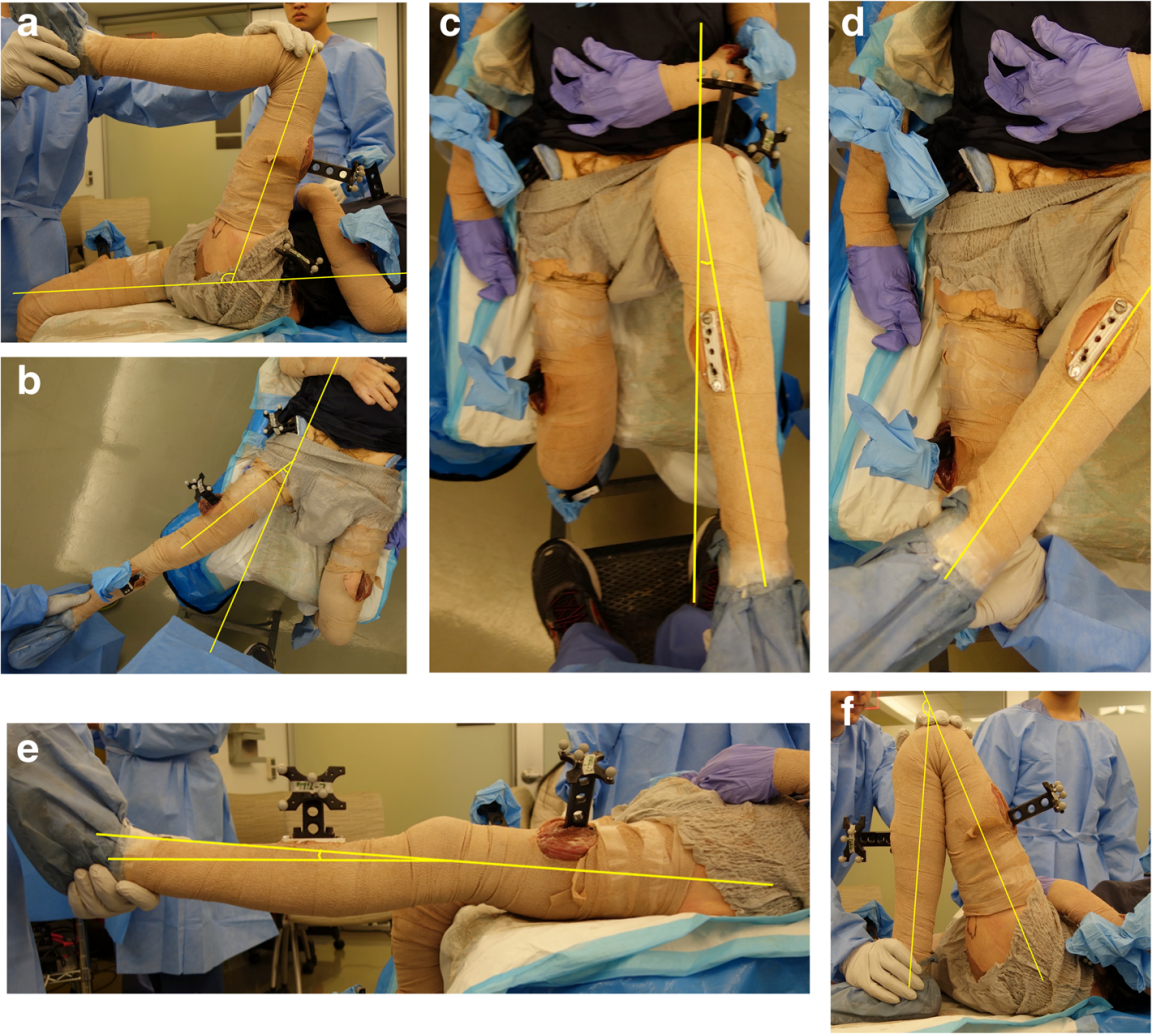
Examples of the investigator’s view during visual estimation, photographing of the limb position (for subsequent angle measurement), and goniometric measurement. All measured positions are shown, including: **a** hip flexion, **b** hip abduction, **c** hip internal rotation, **d** hip external rotation, **e** knee extension, and **f** knee flexion. A stepladder was used when necessary to obtain a “bird’s eye” view of the joint (i.e. hip abduction, internal rotation, and external rotation)

Second, while the assistant held the limb, the investigator would stand three feet from the joint in question at a standardized position (depending on the joint and motion being measured) and make a visual estimation of the joint angle based on each measurers’ estimation of the underlying bone axis of each long bone (as demonstrated in Fig. 3). This distance was chosen as it has been utilized in prior digital photography studies and offered adequate visualization of the bone long axes for all joints (Bennett et al. 2009; Naylor et al. 2011). Third, the investigator would take a digital photograph of the joint angle using a Sony Alpha DSLR-A100 10.2 Megapixel digital camera (Sony Corporation, Tokyo, Japan), but without using a tripod. Finally, a standard plastic goniometer (Patterson Medical, Warrenville, Illinois, USA) was used to measure the joint angle without blinding of the investigator.

Overall, 120 measurements were obtained for each joint motion using each of the three techniques. This was repeated for (A) hip flexion, (B) hip abduction, (C) hip internal rotation, (D) hip external rotation, (E) knee extension, and (F) knee flexion (see Fig. 3). Digital photographs were taken perpendicular to the axis of rotation. The camera was aimed lateral-to-medial (relative to the cadaver) at the hip joint (for hip flexion) and at the knee joint line (for knee extension, and flexion). The camera from anterior-to-posterior (relative to the cadaver) through the hip joint while the investigator stood on a ladder aiming down towards the floor (for hip abduction, internal rotation, and external rotation). These measurements were done bilaterally on each cadaver and repeated for all five cadavers during each of two sessions (total of 10 cadavers measured bilaterally). Digital photographs of each joint were reviewed after the cadaver measurement session, where joint angles were measured using Image J digital measurement software (National Institutes of Health, Bethesda, Maryland) on a 20-in. liquid crystal display computer screen (Dell, Round Rock, TX). ImageJ is free, publically available, Java-based image-processing software designed by the National Institutes of Health (NIH), which allows the angle between two straight lines to be measured on multiple image formats. Lines were drawn as demonstrated in Fig. 3 for each of the six motions described.

### Statistical analysis

Motion capture analysis was used as the “gold standard” with which all measurements by the six investigators using three different techniques (i.e. visual estimation, digital photography, and goniometry) were compared. For analysis, the measurements of all six investigators were combined. *Accuracy* was defined by the authors as the mean measurement error (defined as absolute value of the difference between measurement and gold standard) and was compared between the three measurement techniques. These comparisons were made using analysis of variance (ANOVA) for significant differences between each measurement technique for each hip or knee motion individually. If significant differences were found by ANOVA, a Tukey post-hoc test was performed to identify subgroups with significant differences. ANOVA results are reported along with the degrees of freedom, F-statistic, and statistical significance.

The *precision* of measurements was defined as the proportion of measurement errors less than the minimally clinically important difference (MCID) (Edwards et al. 2004; Gajdosik and Bohannon 1987). These proportions were compared by measurement technique using a Chi squared test (MedCalc, Ostend, Belgium). Statistical significance was defined by an α-value <0.05. The authors chose 10° as the minimal clinically important difference (MCID), or clinically significant difference, for all measurements with the exception of knee extension. Five degrees (5°) was chosen as the MCID for knee extension because less loss of motion would be tolerated clinically with flexion contractures at the knee joint (Edwards et al. 2004; Gajdosik and Bohannon 1987).

## Results

### Hip range of motion (flexion, abduction, internal rotation, external rotation)

There was no significant difference in measurement error between measurement techniques for hip flexion (F(2355) = 2.32; *p* = 0.100), hip internal rotation (F(2354) = 1.97; *p* = 0.140), or hip external rotation (F(2356) = 2.13; *p* = 0.121), shown in Table 1. There was a significant difference in measurement error for hip abduction (F(2357) = 4.18; *p* = 0.016). A Tukey post-hoc test for hip abduction revealed that digital photographic (4.8° ±3.8) measurement had a significantly lower measurement error than visual estimation (6.2° ±4.1, *p* = 0.015).

**Table 1:** Accuracy (Measurement Error) by Technique for Hip Range of Motion

	Visual estimation (Mean ± SD^a^)	Goniometry (Mean ± SD)	Digital photography (Mean ± SD)
Hip Flexion	3.9° ±3.4	3.1° ±2.5	3.5° ±2.7
Hip Abduction	6.2° ±4.1	5.8° ±3.7	4.8° ±3.8
Hip Internal Rotation	7.3° ±5.7	6.8° ±5.1	5.9° ±4.8
Hip External Rotation	10.1° ±6.7	9.7° ±6.0	8.6° ±5.1

Accuracy of measurements (measurement error, in degrees) calculated as the absolute value of the difference between the measurement taken by the investigator using each technique (visual estimation, goniometry, digital photography) and the reference standard (motion capture analysis) for all four hip motions

^a^ *SD* standard deviation

When comparing the proportion of measurements with measurements errors greater than 10° (Table 2), the only significant differences identified were that hip abduction was more precisely measured with digital photography than visual estimation (93% vs. 83%, *p* = 0.019).

**Table 2:** Precision by technique for hip range of motion (proportions of measurement errors within 10° of motion capture analysis)

	Visual estimation	Goniometry	Digital photography
Hip Flexion	91%	96%	96%
Hip Abduction	83%	88%	93%
Hip Internal Rotation	76%	75%	85%
Hip External Rotation	58%	53%	61%

Precision of measurements or the proportion of measurement errors that were within 10°, defined as the minimally clinically significant difference by the authors, of the reference standard (motion capture analysis) for all four hip motions

### Knee ROM (flexion, extension)

There was a significant difference in measurement error for knee flexion (F(2356) = 3.17; *p* = 0.043) and for knee extension (F(2347) = 15.95; *p* < 0.001), shown in Table 3. However, a Tukey post-hoc test for knee flexion did not reveal any significant comparisons (*p* > 0.05). A Tukey post-hoc test for knee extension revealed that digital photographic (3.5° ±2.3) measurement had a significantly lower measurement error than visual estimation (4.7° ±2.5, *p* = 0.001) and goniometry (5.2° ±2.6, *p* = 0.001).

**Table 3:** Accuracy (measurement error) by technique for knee range of motion

	Visual estimation (Mean ± SD^a^)	Goniometry (Mean ± SD)	Digital photography (Mean ± SD)
Knee Flexion	5.2° ±3.9	4.3° ±3.1	5.2° ±3.4
Knee Extension	5.2° ±2.6	4.7° ±2.5	3.5° ±2.3

Accuracy of measurements (measurement error, in degrees) calculated as the absolute value of the difference between the measurement taken by the investigator using each technique (visual estimation, goniometry, digital photography) and the reference standard (motion capture analysis) for both knee motions

^a^ *SD* standard deviation

When comparing the proportion of measurements with measurements errors within the defined clinically significant difference (Table 4), the only significant difference identified was that knee extension was more precisely measured with digital photography than with visual estimation (74% vs. 49%, *p* < 0.001) and with goniometry (74% vs 50%, *p* < 0.001).

**Table 4:** Precision by technique for knee range of motion (proportions of measurement errors within 10° or 5° of motion capture analysis)

	Visual	Goniometer	Photo
Knee Flexion (<10°)	92%	94%	90%
Knee Extension (<5°)	49%	50%	74%

Precision of measurements or the proportion of measurement errors that were within 10° (for elbow flexion) or 5° (for elbow extension), defined as the minimally clinically significant difference by the authors, of the reference standard (motion capture analysis) for both knee motions

## Discussion

The authors hypothesized that digital photography would be equivalent in accuracy and show higher precision compared to visual estimation and goniometric measurement when measuring motion at the hip and knee. Overall, there were only two statistically significant differences in measurement accuracy found with digital photography showing higher accuracy than: (i) visual estimation for hip abduction, and (ii) visual estimation and goniometry for knee extension. Neither of these statistical differences met the authors’ definition of clinical significance confirming the equivalent accuracy of digital photography (compared to goniometry and visual estimation). The maximum difference in measurement error between the three techniques was 1.4° for hip abduction and 1.7° for knee extension. Digital photography proved to have higher precision only for two motions (hip abduction & knee extension) compared to visual estimation and one motion (knee extension) compared to goniometry. Thus, overall digital photography shows equivalent accuracy and precision to visual estimation and goniometry, except for measurements of hip abduction & knee extension. Many studies look specifically at the accuracy and/or reliability of one technique or motion without comparing two or more techniques or motions (Ferriero et al. 2013; Krause et al. 2015; Naylor et al. 2011). Few studies have looked specifically at visual estimation of hip or knee motion (Edwards et al. 2004; Holm et al. 2000; Rachkidi et al. 2009). Edwards et al. found higher accuracy with goniometry compared to visual estimation with 22% and 46% of measurements being within 5° of their gold standard (radiography) for knee flexion only (Edwards et al. 2004). Murphy et al. showed equivalent accuracy of digital photography and goniometry in measuring knee flexion and extension (Murphy et al. 2013). Some studies report an advantage to either digital photography or goniometry over visual estimation with increasing amounts of knee flexion (Ferriero et al. 2013).

Visual estimation is the most common modality used in most surgical practices, due to its speed, ease of use, and lack of need for equipment (Chevillotte et al. 2009; Murphy et al. 2013). The next most common technique, and most commonly used technique among therapists, is goniometry, which is believed by some to offer a more reliable measurement (Ferriero et al. 2013; Gajdosik and Bohannon 1987; Lavernia et al. 2008; Murphy et al. 2013; Watkins et al. 1991). Our study contests that notion with clinically equivalent accuracy between the three techniques. However, digital photography still offered slightly improved precision for measuring hip abduction and knee extension. In addition, digital photography offers the added benefit of a permanent, savable, and printable record of the motion allowing comparison between observations of the same measurement, different measurements separated by time, and allows for off-site measurements over long distances (Bennett et al. 2009; Dunlevy et al. 2005; Ferriero et al. 2013). The ability to accurately measure motion at distance could help facilitate telemedicine or internet-based healthcare, which could alert the clinician regarding declines in function that would benefit from intervention. Often, especially in the hip, motion is included as part of clinical outcome scores (Holm et al. 2000). The ability to obtain digital measures of motion over a distance (by phone or internet) offers great promise for clinical research (Holm et al. 2000). Jenny et al. demonstrated high measurement accuracy at the knee using a smartphone digital camera measurement (Jenny et al. 2016). In this study, we have used a digital camera and secondarily measured the angle on a desktop computer. Although not utilized for this study, smartphone applications allow for identical techniques to be used without the need for transfer of the image to a desktop computer (Ferriero et al. 2013; Milani et al. 2014). This may make digital photographic measurements more clinically attractive alternative to visual estimation or goniometry (Ferriero et al. 2013; Milani et al. 2014).

This study does have some limitations. First, the limb position used for each measurement by each investigator was not identical so comparison of accuracy between measurements relies on the accuracy of the motion capture analysis. Prior studies have shown motion capture analysis to be highly accurate for joint motion measurements making it ideal for use as a gold standard (Charlton et al. 2015; Furtado et al. 2013) and our study utilized arrays of reflective markers that were attached directly to the bones and secured with cement to decrease the possibility of loosening (Chung and Ng 2012). Hagio et al. used CT scans combined with infrared motion capture analysis (similar to this study) and showed excellent accuracy (within 5 degrees) for hip motion (Hagio et al. 2004). Second, motion capsule analysis measures the angle formed by the two bones being measured, which may not represent the “clinical” angle at the joint made by the soft tissue (i.e. with the knee in full extension [or 0°], the bones may be in slight hyper-extension relative to each other). However, this reference remained constant for all measurements by each group allowing comparison between groups. Additionally, other authors have cited radiographic measurement as the “gold standard” which suffers from the same issues (Lavernia et al. 2008). Third, for photographic measurements, we did not measure the distance or angle of the camera in relation to the joint being measured (i.e. no reflective markers were placed onto the camera itself, no use of a tripod or other apparatus). However, this lack of standardization corresponds to the method that it would be used clinically so it allows for better generalization of our results. Fourth, the skin was not marked to identify the optimal points of reference. Instead, each investigator made their own judgment regarding the boney landmarks, in order to be more representative of the clinical utility, which is limited by the amount of body fat, muscle, and clothing obscuring landmarks (Naylor et al. 2011). Again, this will allow better generalization to clinical practice. Fifth, our investigators included three fellowship-trained orthopaedic surgeons and three physical therapists with varied levels of experience. This may have had an effect on the measurement accuracy and reliability. Sixth, the clinical applicability of measurement errors are not static across a range of motion. An error of 5–10° at 100° knee flexion is less clinically significant than that same error at full extension (Ferriero et al. 2013). The definition of clinically significant changes in motion (such as minimal clinically important difference [MCID]; minimal detectable change [MDC]) or minimal acceptable motion (such as patient acceptable symptom state [PASS]) for joint range of motion is not well established within the literature. Some suggest 6° be used for the lower extremity, while others define clinical significance by a change greater than 10% of the motion arc (Blonna et al. 2012; Boone et al. 1978; Mehrholz et al. 2005; Roach et al. 2013; Wheatley-Smith et al. 2013). However, for certain joints, 10% seems excessive (i.e. 14° for knee extension) (Mehrholz et al. 2005; Roach et al. 2013; Wheatley-Smith et al. 2013). The authors chose 10° as the MCID, or clinically significant difference, for all measurements with the exception of knee extension.

## Conclusions

There was no clinically significant difference in measurement accuracy between the three techniques for hip and knee motion. Digital photography only showed higher precision for two joint motions (hip abduction and knee extension). Overall digital photography shows equivalent accuracy and near-equivalent precision to visual estimation and goniometry.
